# Clinical Utility Assessment of a Nursing Checklist Identifying Complex Care Needs Due to Inequities Among Ambulatory Patients With Cancer: Protocol for a Mixed Methods Study

**DOI:** 10.2196/48432

**Published:** 2023-11-09

**Authors:** Holly Chung, Amelia Hyatt, Elizabeth Crone, Donna Milne, Sanchia Aranda, Karla Gough, Meinir Krishnasamy

**Affiliations:** 1 Academic Nursing Unit Peter MacCallum Cancer Centre Melbourne Australia; 2 Department of Health Services Research and Implementation Science Peter MacCallum Cancer Centre Melbourne Australia; 3 Sir Peter MacCallum Department of Oncology University of Melbourne Melbourne Australia; 4 Department of Nursing School of Health Sciences University of Melbourne Melbourne Australia; 5 Skin and Melanoma Service Peter MacCallum Cancer Centre Melbourne Australia; 6 Victorian Comprehensive Cancer Centre Alliance Victoria Australia

**Keywords:** cancer, oncology, cancer nursing, clinical utility, nursing checklist, social determinants of health, equity, cancer care, disparity, barrier, checklist, nursing, nurse, caregiver, specialist nurse, patient outcome

## Abstract

**Background:**

Disparities in cancer incidence, complex care needs, and poor health outcomes are largely driven by structural inequities stemming from social determinants of health. To date, no evidence-based clinical tool has been developed to identify newly diagnosed patients at risk of poorer outcomes. Specialist cancer nurses are well-positioned to ameliorate inequity of opportunity for optimal care, treatment, and outcomes through timely screening, assessment, and intervention. We designed a nursing complexity checklist (the “Checklist”) to support these activities, with the ultimate goal of improving equitable experiences and outcomes of care. This study aims to generate evidence regarding the clinical utility of the Checklist.

**Objective:**

The primary objectives of this study are to provide qualitative evidence regarding key aspects of the Checklist’s clinical utility (appropriateness, acceptability, and practicability), informed by Smart’s multidimensional model of clinical utility. Secondary objectives explore the predictive value of the Checklist and concordance between specific checklist items and patient-reported outcome measures.

**Methods:**

This prospective mixed methods case series study will recruit up to 60 newly diagnosed patients with cancer and 10 specialist nurses from a specialist cancer center. Nurses will complete the Checklist with patient participants. Within 2 weeks of Checklist completion, patients will complete 5 patient-reported outcome measures with established psychometric properties that correspond to specific checklist items and an individual semistructured interview to explore Checklist clinical utility. Interviews with nurses will occur 12 and 24 weeks after they first complete a checklist, exploring perceptions of the Checklist’s clinical utility including barriers and facilitators to implementation. Data describing planned and unplanned patient service use will be collected from patient follow-up interviews at 12 weeks and the electronic medical record at 24 weeks after Checklist completion. Descriptive statistics will summarize operational, checklist, and electronic medical record data. The predictive value of the Checklist and the relationship between specific checklist items and relevant patient-reported outcome measures will be examined using descriptive statistics, contingency tables, measures of association, and plots as appropriate. Qualitative data will be analyzed using a content analysis approach.

**Results:**

This study was approved by the institution’s ethics committee. The enrollment period commenced in May 2022 and ended in November 2022. In total, 37 patients with cancer and 7 specialist cancer nurses were recruited at this time. Data collection is scheduled for completion at the end of May 2023.

**Conclusions:**

This study will evaluate key clinical utility dimensions of a nursing complexity checklist. It will also provide preliminary evidence on its predictive value and information to support its seamless implementation into everyday practice including, but not limited to, possible revisions to the Checklist, instructions, and training for relevant personnel. Future implementation of this Checklist may improve equity of opportunity of access to care for patients with cancer.

**International Registered Report Identifier (IRRID):**

DERR1-10.2196/48432

## Introduction

The past decade saw remarkable advances in cancer care, treatment, and outcomes, but these benefits were not equitably realized. Concerningly, cancer disparities between populations of high and low socioeconomic status continue to widen, and those disadvantaged by social determinants of health experience a disproportionate burden of poorer cancer outcomes [1]. Social determinants of health, such as income and social protection, education, food security, and social inclusion and nondiscrimination, have major impacts on health and health outcomes of populations [2,3]. This is especially true for people affected by cancer, whereby those disadvantaged due to the impact of social determinants experience significantly more disease burden and are more likely to die from their cancer than others [2,4].

A significant proportion of this burden is due to systemic barriers influencing access to diagnosis, treatment, and care. For example, distance from treatment centers, out-of-pocket costs associated with accessing treatment and care, and overly complex health care systems that are difficult to navigate without adequate information and support [5,6]. In countries like Australia, health services inadequately address the requirements of the communities they serve; for example, insufficient provision of information in languages other than English, and institutional racism that particularly affects experiences and outcomes for Aboriginal and Torres Strait Islander patients [7]. The evolution of the concept of the “complex patient” acknowledges that individual patients are not inherently complex, but rather face a complex intersection of interrelating factors on multiple levels: biomedical (diagnoses and comorbidities), individual (language, literacy, and cultural), social (support, inclusion, and discrimination), and environmental (access to food, housing, and transportation) [8]. If unaddressed, these factors may manifest in a higher risk of morbidity, challenges to accessing appropriate health care, receipt of worse care, and consequently worse health outcomes. Although these findings are well recognized, few attempts exist to systematically integrate point-of-care interventions to rapidly identify people requiring early support or increased follow-up to address or mediate access barriers and systemic inequities that maximize health outcomes.

Evidence demonstrates that cancer nurses address critical gaps in care coordination for patients with cancer resulting in improved health outcomes and overall survival [9]. Cancer nurses are therefore ideally placed to identify and address these consequences flowing from structural inequities that impact equitable access to diagnosis, treatment, and care. However, there are considerable and increasing discrepancies between the available cancer nursing workforce and the demand for services, with a recent report published by the World Health Organization estimating a global shortage of 5.7 million nurses by 2030 [10]. There are also policy and funding barriers to innovations in care, such as nurse-led services.

Concurrently, there is a projected 47% increase in cancer burden expected from 2020 to 2040 across all settings globally, though particularly affecting low-resource settings [11]. With growing numbers of patients with cancer without a corresponding increase in specialist nurses, the ability to quickly identify and target those most in need of timely support is critical. Resourcing cancer nurses with validated tools to efficiently and systematically identify people with or at risk of complex care needs presents an overlooked opportunity to redress inequities in cancer care. For the purpose of this study, “complex care needs” are defined as the presence of access barriers to care and increased needs individuals newly diagnosed with cancer face due to the presence of social determinants of health and associated systemic barriers. The Complexity Checklist (the “Checklist”) is a novel cancer nursing checklist developed to facilitate early identification of newly diagnosed patients with cancer at risk of poor experiences and outcomes due to complex care needs. In a review of internationally published papers, no similar checklist exists for oncology settings.

To generate evidence regarding the potential benefit of the Checklist, this study will undertake a comprehensive assessment of the Checklist’s clinical utility. Clinical utility refers to the practical usefulness of an intervention to improve health outcomes. This study is informed by Smart’s multidimensional model of clinical utility, which describes 4 dimensions that impact on an intervention’s clinical utility: appropriateness, accessibility, acceptability, and practicability (Table 1) [12]. “Accessibility” will not be examined as it pertains to economic considerations of an intervention, which is out of the scope of this study. “Appropriateness” explores the evidence of the effectiveness and relevance of an intervention to clinical decision-making, and disruptions to current work and care [12]. Acceptability explores whether the intervention is acceptable to service users, providers, and broader society [12]. “Practicability” examines suitability, whether the intervention performs in real-world settings; functionality, whether the intervention, training, and instructions are complete and in working order; as well as current and future training and knowledge requirements [12].

**Table 1:** Study objectives, type of data, and data source for a mixed methods case series study investigating the clinical utility of a nursing checklist for newly diagnosed patients with cancer in a specialist cancer hospital. Data collection activities occur over a 6-month period.

Study objective	Type of data	Data source
To determine patient and specialist nurse perspectives regarding the relevance or importance of the Checklist for decision-making, and disruptions to current work and care.	Perspectives of patients and CNCs^a^ of ease of use of Checklist and meaning and relevance of information obtained. Time taken to complete the Checklist and make referrals.	Interviews with patients (T1) and CNCs (T2 and T3). CNC postchecklist survey, patient and CNC interviews.
To determine whether the Checklist is acceptable to patients newly diagnosed with cancer and specialist nurses delivering care.	Perspectives of patients and CNCs. Number of patients approached and recruited for the study. Number of Checklists completed at T1.	Interviews with patients and CNCs. Checklist, operational, and EMR^b^ data.
To determine whether the Checklist, training, and instructions are complete and in working order.	Perspectives of patients and CNCs regarding whether the Checklist addresses all issues of importance/relevance to participants.	Interviews with patients and CNCs.
To determine the perspectives of specialist nurses regarding the adequacy of training and current staff knowledge to use the Checklist and future needs.	Perspectives of CNCs on the adequacy of training delivered to use the Checklist in practice and perceptions of future needs.	Interviews with CNCs.
To explore the predictive value of the Checklist.	CNC classification at enrollment and at 24-week follow-up.	Checklist, operational and EMR data (internal and external referrals and internal uptake), and patient interview data (external uptake).
To explore concordance between specific checklist items and PROMs^c^.	Responses to prespecified Checklist items and relevant PROMs scores.	Checklist, PROMs data.

^a^CNC: clinical nurse consultant.

^b^EMR: electronic medical record.

^c^PROMs: patient-reported outcome measures.

This study aims to provide an assessment of the clinical utility of the Checklist. Perspectives of newly diagnosed patients with cancer and specialist nurses will be used to explore the following 4 primary objectives:

- To determine patient and specialist nurse perspectives regarding the relevance or importance of the Checklist for decision-making, and disruptions to current work and care.
- To determine whether the Checklist is acceptable to patients newly diagnosed with cancer and specialist nurses delivering care.
- To determine whether the Checklist, training, and instructions are complete and in working order.
- To determine qualitative perspectives of specialist nurses regarding the adequacy of training and current staff knowledge to use the Checklist and future needs.

In addition, 2 secondary objectives have also been identified:

- To explore the predictive value of the Checklist.
- To explore concordance between specific checklist items and patient-reported outcome measures (PROMs).

## Methods

### Study Design and Setting

This prospective mixed methods case series study was designed to investigate the clinical utility of a nursing complexity checklist. The study will be conducted in accordance with the Joanna Briggs Checklist for Case Series [13]. Specialist nurses will complete the Checklist with consecutive patients newly diagnosed with cancer; then quantitative and qualitative data will be collected multiple times following completion of the Checklist at enrollment up to 24 weeks post Checklist completion. The study will be conducted at a specialist cancer center in Victoria, Australia. Ethical approval was granted by the institutional ethics committee (HREC/84219/PMCC).

### Development

**Figure 1:**
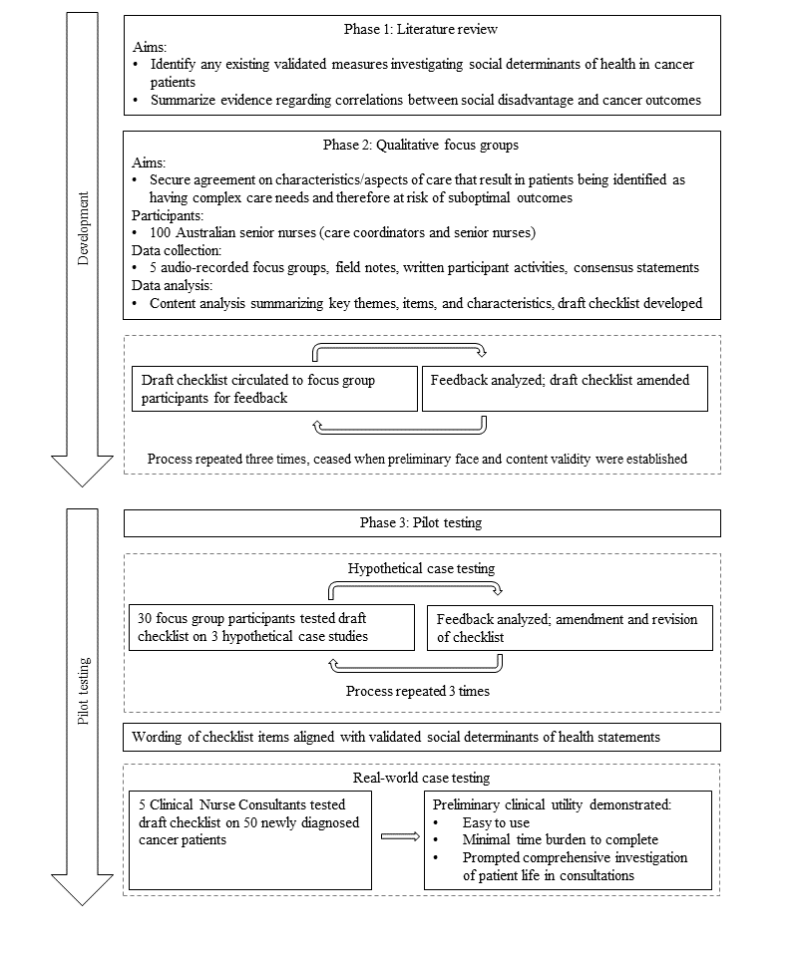
Development of a nursing checklist to assess patients with cancer for risk of complex care needs with senior cancer nurses in Australia, 2016-2019.

The Checklist was developed between 2016 and 2019 by members of our team (MK, DM, AH, and KG; Figure 1). A comprehensive review of published literature was undertaken to identify existing measures investigating social determinants of health in patients with cancer and to summarize evidence on associations between social disadvantage and cancer outcomes. Next, 100 Australian cancer nurses from Victoria, New South Wales, Queensland, South Australia, and Western Australia participated in 5 qualitative focus group consultations conducted face-to-face and digitally. Focus groups aimed to secure agreement on characteristics or aspects of care that result in a patient having complex care coordination needs likely to increase their risk of suboptimal outcomes. Audio-recorded and written qualitative data were analyzed using content analysis. The first iteration of the Checklist was developed and circulated to nurse participants for feedback. This process occurred 3 times until no new characteristics or aspects of care were proposed for inclusion (preliminary content validity) and participants felt items included were easily understood (preliminary face validity).

The Checklist was then pilot-tested through hypothetical and real-world case testing. In total, 30 nurses (drawn from focus group participants) tested the Checklist with 3 hypothetical case studies to provide data on ease of use, and interpretation of the items when applied to a patient case. Feedback guided amendment and revision of the number of items included in the Checklist (for example, items were collapsed or excluded as repetitive) over 3 iterative cycles until no further modifications were required. Following the establishment of Checklist constructs, a series of study team workshops were undertaken to operationalize constructs identified in the nurse workshops into quantitively measurable index items. Items were aligned items with existing psychometric and clinical assessments, as well as standardized definitions to enhance face and construct validity. The revised Checklist was then tested by 5 Clinical Nurse Consultants (CNCs) at a specialist cancer hospital in Australia who each used the Checklist in real-world consultations with 10 newly referred patients with cancer. Pilot-testing found the Checklist to be easy to use, took minimal time to complete, and prompted CNCs to ask their patients questions they may not otherwise have done.

### Description

**Figure 2:**
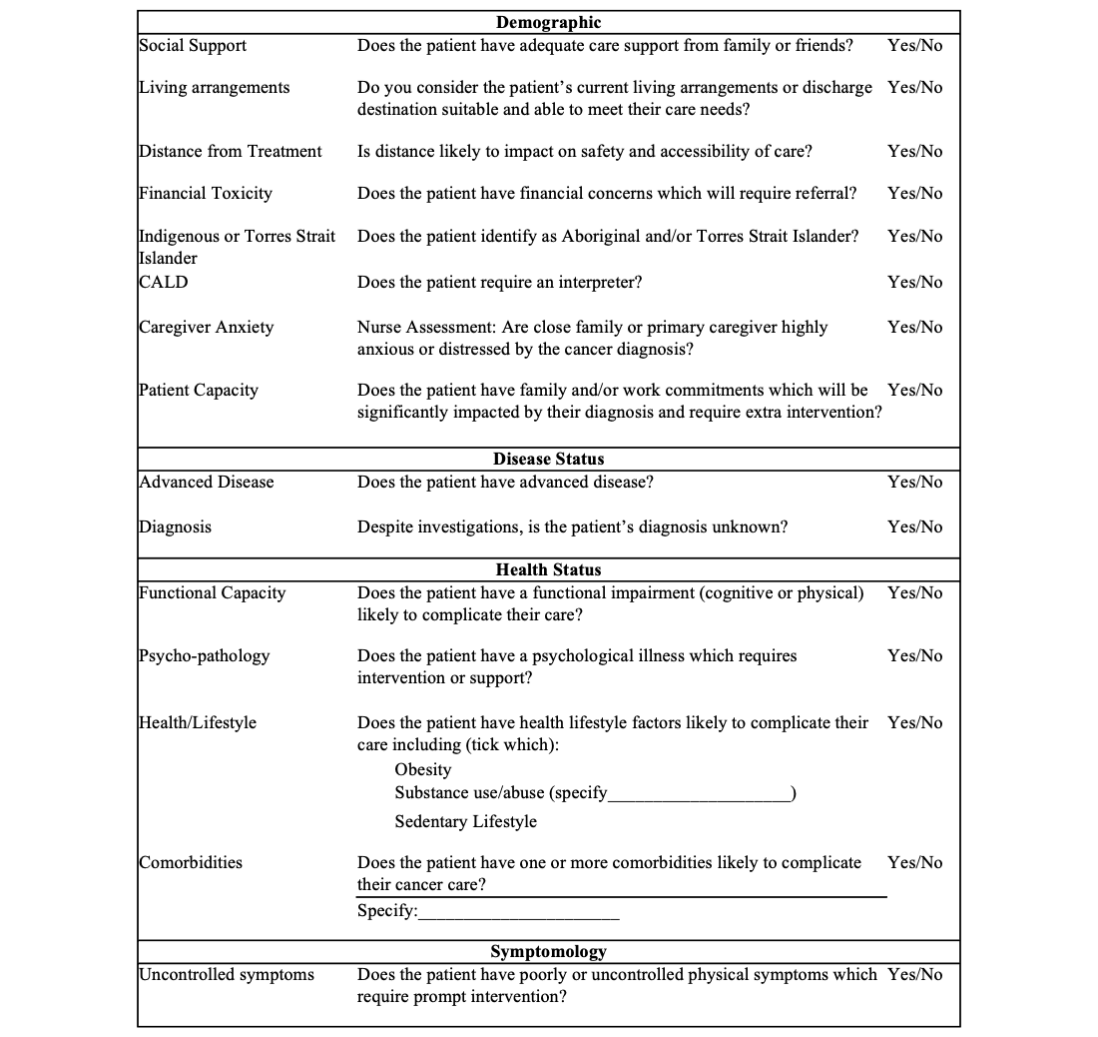
The Complexity Checklist, a novel nursing checklist to identify patients with cancer at risk of complex care needs developed in Australia 2016-2019.

The Checklist comprises 15 items mapping to 4 domains: demographic, disease, health status, and symptomatology (Figure 2). Items are answered using a yes/no response format. The Checklist has been designed as a tool to prompt systematic assessment of newly diagnosed patients with cancer during consultations. This is achieved by guiding nurses through a comprehensive clinical consultation, to elicit all relevant information on factors that may add to a person’s experiences of complexity. Data generated by the Checklist will be used by the CNCs to initiate referrals and with other members of the treating team; for example, through the electronic medical record or multidisciplinary team meetings.

### CNC Training

Nurses will participate in a training session on the use of the Checklist to optimize generalizability across observers (or raters). The session will provide guidance for specific items such as recommended daily exercise and guidelines regarding safe alcohol intake [14,15].

### Patient and Public Involvement

This study builds upon preliminary clinical utility data from 5 CNCs who used the Checklist with 50 newly diagnosed patients with cancer. In addition, 2 consumer representatives were appointed to the study’s steering committee following protocol development and have assisted with guiding the study amendments and direction.

### Participants

A consecutive sample of newly diagnosed patients with cancer from 4 cancer streams (gynecological, urological, head and neck, and lung cancers) will be recruited. In accordance with the equity focus of the Checklist, support to enable participation of non-English speaking patients is provided. As this study investigates care delivery, the study targets people with a new diagnosis of cancer or where a previous diagnosis was managed with a simple excision. Patients attending the health service solely for the provision of clinical trial access are also excluded as trial care is not standard of care. Potential patient participants will be identified by participating CNCs from clinic and referral triage lists. A project officer (PO) will screen identified patients according to the following eligibility criteria:

- are 18 years or older
- have a histologically confirmed cancer diagnosis
- are expected to survive at least 24 weeks from the time of recruitment, as determined by tumor stream CNC
- are referred to the head and neck, gynecological, urological, or lung treating teams at the hospital for major surgery, more than a single fraction of radiotherapy or systemic therapy.
- are approached to participate within a 4-week window of their first appointment at the hospital or are approached within 4 weeks following a confirmed cancer diagnosis (for patients referred for diagnostic workup)
- are able to complete Checklist-related questions and take part in an audio-recorded semistructured interview either independently or with support (such as a caregiver, family member, or interpreter) as per usual clinical care
- are able to give informed consent
- have no previous cancer diagnosis (except basal cell carcinoma and squamous cell carcinoma type skin cancers and in situ carcinomas)
- have not been referred to Peter Mac solely to access treatment through a clinical trial

Eligible patients will be approached via telephone to introduce the study and invite participation via telephone. Interested eligible participants will be requested to provide written informed consent. CNCs from each participating cancer stream will be eligible to participate in the study. A maximum of 5 CNCs from each cancer stream will be recruited, to ensure all CNCs gain familiarity with delivering the tool.

### Ethical Considerations

This study protocol has been approved by the relevant institution’s Human Research Ethics Committee (22/39L). Written informed consent will be requested from all participants prior to study enrollment. Verbal reconfirmation of consent is audio-recorded at the beginning of semistructured interviews with all CNC and patient participants. Participants will not receive compensation for their involvement in this study. Participant data will be stored in accordance with National Health and Medical Research Council guidelines and deidentified where possible (refer to data management below for further detail).

### Study Size

The study will aim to recruit 15 patients from each participating cancer stream (60 patient participants in total), and a minimum of 1 to a maximum of 5 CNCs working in each of the 4 participating cancer streams. The sample size is pragmatic based on study timeframes, clinical throughput and funds available These sample sizes will provide adequate information to achieve the primary study objectives, explored through qualitative interviews with patients and CNCs [16].

### Data Sources and Measurement

**Figure 3:**
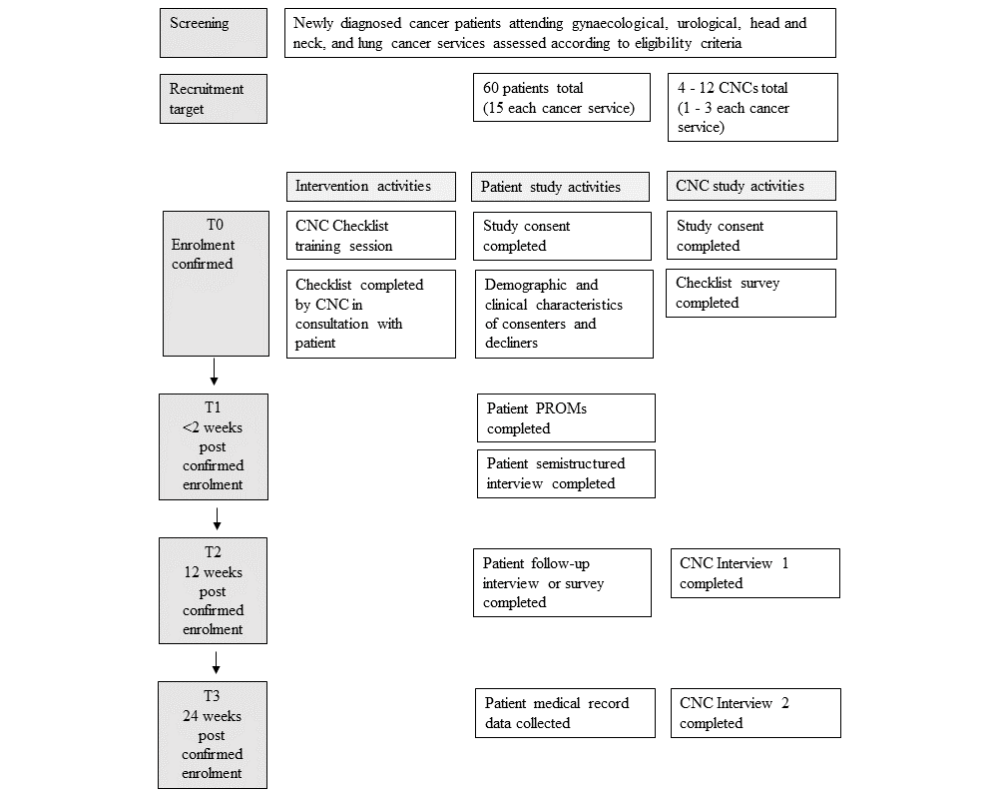
Study schema for a mixed methods case-series study investigating the clinical utility of a nursing checklist for newly diagnosed patients with cancer in a specialist cancer hospital. Data collection activities occur over a 6-month period. CNC: clinical nurse consultant; PROM: patient-reported outcome measure.

Data collection activities will occur at 4 timepoints (Figure 3). Study enrollment of patient participants is confirmed once the intervention (the Checklist) is completed with their CNC (T0). Study enrollment for CNC participants is similarly confirmed the first time they complete a Checklist with a patient participant. Patients will complete 5 PROMs and an audio-recorded semistructured interview within 2 weeks of completing the checklist (T1), and a follow-up interview at 12 weeks post completion (T2). CNCs will complete 2 qualitative semistructured interviews at 12 weeks (T2) and 24 weeks (T3) after completing their first checklist. Medical record data to describe each patient’s care events across the 24-week data collection period will be collected at T3. Qualitative and quantitative data collected will be used to evaluate multiple aspects of the clinical utility of the Checklist (Table 2).

**Table 2:** Checklist items and corresponding PROMs^a^ used to investigate concordance in a mixed methods case series study at a specialist cancer hospital.

Complexity Checklist item	Corresponding PROM
1) Social support	PROMIS^b^ Instrumental Support SF^c^ [17]
4) Financial toxicity	COST-FACIT [18]
8) Patient capacity	PROMIS ability to participate in social roles and activities—SF [17]
11) Functional capacity	PROMIS physical function SF [17]
15) Uncontrolled symptoms	PROMIS self-efficacy for managing symptoms SF [17]

^a^PROM: patient-reported outcome measure.

^b^PROMIS: patient-reported outcomes measurement information system.

^c^SF: short form.

### T0 Data Collection Activities and Procedures

#### Operational Data

Details of patients screened, approached, consented, and declined will be entered into a password-protected Excel (Microsoft Corp) spreadsheet, hosted on a secure hospital server.

#### Patient Information

Demographic, disease, and treatment-related data will be extracted from the electronic medical record (EMR) by members of the project team (HC and EC) for all patient participants (consenters). Data will include sex, age, marital status, country of birth, primary language spoken, postcode, living situation, disease type and stage, comorbidities, treatment plan, and treatment intent and entered into Research Electronic Data Capture (REDCap; Vanderbilt University)—a web-based electronic data capture tool hosted by the hospital [19,20]. Demographic and disease data will also be extracted from the EMR for all eligible participants who decline to participate (decliners) and entered into a separate REDCap database.

#### CNC Postchecklist Survey

CNCs will complete a paper-based or electronic checklist with patient participants. After completing each checklist, CNCs will also complete a study-specific survey to record: whether the checklist was completed face-to-face or digitally (telephone or telehealth); whether and from who the patient received assistance answering checklist items; and whether the CNC deemed the patient as having complex care needs based on Checklist items (response options: complex, not complex, and unsure). Checklist and CNC survey data will be entered into a web-based REDCap database by a study PO.

### T1 Data Collection Activities

#### Initial Patient Interview

Patient participants will take part in an audio-recorded semistructured interview investigating patients’ experiences and views regarding the acceptability, appropriateness, and practicability of the Checklist. Interviews will be conducted by a study PO face-to-face, by telephone, or via videoconferencing as per participant preference, and will take approximately 20-30 minutes to complete.

#### PROMs

Patient participants will complete 5 PROMs at the beginning of their semistructured interview (listed in Table 2). PROM data will be entered into a web-based REDCap database by a study PO. Four PROMS are drawn from the Patient-Reported Outcomes Measurement Information System, a system of widely used, highly reliable, and precise measures of patient-reported health status for physical, mental, and social well-being [17]. The fifth measure is the COST-FACIT scale, an 11-item measure of the impacts of chronic illness and its treatment on financial status [18].

### T2 Data Collection Activities

#### Patient Follow-Up Interview

Approximately 12 weeks after administration of the Checklist, patient participants will be invited to complete a short follow-up interview. The purpose of this is to understand each patient’s pattern of service use regarding uptake of internal and external referrals made at and following T1, and unplanned service use, particularly external care not captured by the hospital EMR.

#### Initial CNC Interview

CNCs will participate in an audio-recorded semistructured interview 12 weeks after they first administered a Checklist. This interview will explore the CNCs’ perspectives on the appropriateness, acceptability, practicability, and clinical application of the Checklist. Demographic data will be collected at the start of interviews and comprise role title and discipline, years working at Peter Mac, and cancer stream affiliation. Interviews will be conducted face-to-face or by videoconferences as per participant preference and will take approximately 45-60 minutes to complete.

### T3 Data Collection Activities

#### CNC Follow-Up Interview

Approximately 24 weeks after the first Checklist administration, CNCs will participate in a second audio-recorded semistructured interview. The purpose of this interview is to explore CNC perceptions of the discriminatory capacity of the Checklist, as well as its clinical utility and application. Additionally, checklist, operational and EMR data combined with CNC judgment will be used to determine complex care needs “caseness” at 24 weeks after enrollment. Interviews will be conducted as per participant preference and take approximately 20-30 minutes to complete.

#### Patient EMR Data

POs will complete a review of the patient’s EMR to record treatment-related outcomes 24 weeks after patient participants complete the Checklist. These data include whether treatment was completed, amended, or the patient has withdrawn from treatment, transitioned to a clinical trial, or transitioned to palliative care. Referral data will also be collected, to add to and cross-check patient T2 follow-up. Data will capture types and dates of internal and external referrals, unplanned contacts with nurses and other health care professionals, and information regarding the uptake of internal referrals.

### Data Analysis Plan

#### Qualitative Data

Audio-recorded interviews will be translated (as required), transcribed verbatim, and deidentified. NVivo 12 (QSR International) will be used to assist with data management. Three distinct tranches of semistructured interview data (patient T1, CNC T2, and CNC T3) will be analyzed separately using deductive and inductive content analysis. Content analysis allows for systematic and objective descriptions of phenomena such as views and perceptions of participants and is well-suited to informing clinical improvements [21].

An initial deductive approach will operationalize the prespecified study objectives (Table 1) to construct an initial, unconstrained, coding frame for each tranche of interviews. The coding frames will be tested and inductively revised to classify data into relevant codes and categories under clinical utility themes. In addition, 2 POs (HC and EC) trained in qualitative research will independently code 10% of each interview tranche to test and revise the coding frames. Iterations of the coding frames will be discussed, and discrepancies within coding frames will be resolved by consensus. The project principal investigator (MK) will be assigned a “third reviewer” role if HC and EC are unable to resolve discrepancies. Upon conclusion of the qualitative analysis, findings (codebooks and summaries) will be presented and discussed with members of the study team. Any divergence or disagreement with interpretation will be resolved by consensus. Following the completion of coding frames, qualitative data will be synthesized to explore similarities and disagreements between participant cohort perspectives regarding the clinical utility of the Checklist.

#### Quantitative Data

Data will be exported from REDCap, and then imported into R for scoring and analysis (reference index version 4.2.3 or higher). PROMs will be scored according to author guidelines and standard recodes applied as required [22-27]. T0 “caseness” classifications will be recoded to a discrete variable comprising 2 categories: case or noncase (noncase and unsure).

Counts and percentages will be used to summarize missing operational, participant characteristics (patient and CNC), checklist, EMR, and PROMs data. Missing data will not be imputed. Descriptive statistics (counts and percentages, means and SDs, medians and IQRs, and ranges, as appropriate) will be used to summarize the characteristics of patient consenters and decliners for the full sample and by cancer service, as well as the demographic and professional characteristics of CNC participants. Counts and percentages will be used to summarize “caseness” data (complex, not complex, unsure) from the CNC survey completed at enrollment. Contingency tables and conditional probabilities will be used to explore the predictive value of the Checklist by assessing agreement between T0 and T3 “caseness” variables. Descriptive statistics, measures of association, and graphical displays will be used to examine the concordance between prespecified checklist items and PROMs scores [28].

### Data Management

#### Data Storage and Access

Demographic, Checklist, and PROMs data will be recorded onto hard-copy paper forms and entered into a study database developed and stored on the hospital REDCap platform. All study data (such as REDCap exports, qualitative data files, and analysis files) will be stored securely in password-protected electronic folders, housed on a secure hospital server. Quality checks will be undertaken at regular intervals by POs during data collection to ensure completeness, precision, and timeliness of data. Access to all hard-copy and electronic data will be restricted to the project principal investigator and POs directly involved in data collection and analysis.

#### Privacy

Study identification codes will be assigned to each participant and decliner and used to label data collected to protect privacy. Separate master lists, linking study identification codes to identifiable information (such as demographic and contact data) will be constructed for participants and decliners. Separate REDCap databases will be developed to store the participant master list, decliner master list, and Complexity study data. As above, only key project staff will be given access to these databases.

#### Data Retention and Disposal

At study closure, REDCap databases will be archived from the platform. Password-protected exports of all databases will be saved onto the secure hospital server, and hard-copy data placed into secure storage. All study data will be stored in accordance with National Health and Medical Research Council guidelines for the conduct of research for the duration of the study and 5 years post publication, in accordance with best practice research guidelines [29].

## Results

This study received a grant from Perpetual’s IMPACT Philanthropy Application Program in 2021 and was approved by the Peter MacCallum Cancer Centre Human Research Ethics Council in May 2022 (HREC/84219/PMCC). In total, 37 patient participants and 7 CNC participants were recruited across gynecological, head and neck, lung, and urological cancer streams from May 10, 2022, to October 19, 2022. Data collection, cleaning, and analysis activities are ongoing, and data collection will be concluded in April 2023. Study results are expected to be disseminated by the end of 2023.

## Discussion

### Principal Findings

This study will provide a comprehensive assessment of the Complexity Checklist’s acceptability, appropriateness, and practicability (as defined by Smarts’ multidimensional model of clinical utility) [12]. These data will provide understanding regarding patient and CNC perspectives on the Checklist’s clinical utility and inform recommendations for changes to the Checklist (addition, removal, and modifications of items), and changes to training and instructions provided to clinicians delivering the Checklist. Findings from exploratory objectives will give early evidence of the Checklist’s predictive capability and concordance with evidence-based measures. These findings will guide Checklist refinement, as well as justification and development of future studies assessing the Checklist’s predictive capability and potential to impact clinical outcomes

Recent literature promotes the importance of understanding the impacts of social determinants of health (SDoH) on clinical care, though the definition and inclusion of SDoH in previous clinical checklists are varied [1,30]. The Complexity Checklist comprises structural (gender, education, and socioeconomic position) and intermediary determinants (psychosocial, behavioral, and biological factors), as well as individual medical factors that senior cancer nurses identified as contributing to worse patient experiences and outcomes [3]. This checklist has the potential to serve dual purposes: informing care for individual patients, while also gathering structured data that will enrich health service understanding of the distribution and impact of SDoH within their patient population to inform system and service level change.

### Limitations

This mixed methods study is not sufficiently powered to establish statistical evidence for the Checklist, though the target sample size is suitable to inform qualitative findings. The case-series approach, with no control group, aims to describe the characteristics and outcomes of patient participants rather than investigate the effectiveness of the intervention. These limitations will be addressed in a future large-scale trial if this study supports justification for further investigation. Recruitment will occur at a specialist metropolitan cancer hospital in Victoria, and a lack of diversity in patient participants may result. However, inclusion criteria have been developed to enable participation from non-English speaking persons. Future studies will specifically explore the clinical utility of the Checklist in culturally diverse cancer populations, acknowledging the importance of constructing cross-culturally valid measures [31].

### Dissemination

Following data analysis, findings will be presented to the study funder as required by the funding agreement. Findings will be disseminated through standard scientific channels including peer-reviewed publications, conference presentations, and communities of practice, as well as to a broader audience including media, government, service users, and clinicians. The study investigators have a strong interest in promoting awareness of how social disadvantage affects cancer experiences and outcomes, and how inequities in cancer care can be addressed.

### Conclusions

Structural and intermediatory determinants of health can result in access barriers to health care, posing a significant risk to achieving equity in cancer care. The Checklist aims to assist nurses to quickly and in a standardized manner, identify people disadvantaged due to social determinants of health at risk of complex care needs, so necessary supports can be provided. Pilot-testing had shown early evidence of potential. The study outlined above will assess the acceptability, appropriateness, and practicability of the measure, from the perspectives of newly diagnosed patients with cancer and specialist nurses involved in their care. Findings will inform further development and future studies to assess the Checklist. Integration of the Checklist into routine care offers the opportunity to improve patient care and outcomes, as well as create data sets with information regarding the SDoH to inform health service reform.

## Abbreviations

CNC: clinical nurse consultant
EMR: electronic medical record
PO: project officer
PROM: patient-reported outcome measure
REDCap: Research Electronic Data Capture
SDoH: social determinants of health

## Data Availability

Data generated by this study will be available from the corresponding author upon reasonable request.
